# Glances at the Hospitals

**Published:** 1901-11-23

**Authors:** 


### GLANCES AT THE HOSPITALS.

### MERCER'S HOSPITAL, DUBLIN.

The income was £2,734 and the expenditure £3,379, leaving a balance of £638 due to the National Bank. The subscriptions were nearly £100 lower than in the previous year, and about 3,000 additional out-patients have been treated. The Governors say they "rely on the benevolence of the public," and that they will "exercise the strictest economy without reducing the amount of relief." We hope to hear from next year's report that the Governors' reliance on the public benevolence has not been misplaced, and that the debt has been wiped off. At the beginning of the year there were 61 patients in residence, and 1,016 were admitted during the year, making a total of 1,077 under treatment. In the out-patients' department there were 18,400 attendances, making a grand total of 19,477 patients during the year. The operations table shows that 80 operations were performed on the head and neck with 2 deaths, 17 on the thorax with 1 death, 144 on the abdomen with 3 deaths, 126 on the upper extremity, and 73 on the lower extremity without any death. The mortality after operations was only 1·363 per cent. The total number of deaths during the year was 28 in the medical wards and 19 in the surgical. The former gives a percentage of 5·70, and the latter of 3·31. A medical table might be advantageously added next year, and the surgical tables made a little fuller. Also an anæsthetic table should be given.

### ADDENBROOKE'S HOSPITAL, CAMBRIDGE.

The receipts were £6,911, and the expenditure was £7,895. There is a balance of £2,373 due to the treasurer. The total number of beds is 170, of which number 23 are devoted to children. The Cambridge Medical School has a connection with the hospital, and the University has increased its annual contribution from £50 to £300. The total number of in-patients was 1,546, and of these 558 were medical cases, 927 surgical, and 61 were ophthalmic. The out-patients numbered 5,381. The Nurses' Training School was established in the year 1877. In those early days of scientific nursing one year's training was considered sufficient, but now three years are not thought too much, and no certificate at Cambridge is given for a shorter time. The probationers pay £40 for the first year, £20 for the second, and the third is free. Last year these fees amounted to £814, so that a training school is not an unprofitable adjunct to a hospital. There are in this report 75 pages of names of subscribers and contributors, but not a word is said about the patients treated during the year, although the fact is emphasised that there is a medical school attached to the hospital.

### WEST SUSSEX, EAST HANTS AND CHICHESTER INFIRMARY.

The total cash receipts were £2,610, and the expenditure was £3,316, leaving a balance of £628 due to the treasurer. This adverse balance is accounted for by the increased price of coal and drugs, and by a considerable outlay for the purchase of surgical instruments. However necessary or unavoidable all these expenses may have been £628 is a large sum for a small infirmary to have to become indebted for in one year, and the committee do not say how they mean to deal with the deficit. During the year 448 patients were treated in the infirmary. Of these 315 were cured, 51 relieved, 4 not improved, 38 died, and 40 remained in the house. The nnmber of out-patients was 1,555, and, in addition, 330 were visited at their own homes. This is one of the few hospitals which publishes no medical or surgical statistics.

### ALEXANDRA INFIRMARY, PAISLEY.

The income was £5,502 and the expenditure was £5,818. There is an adverse balance of £836, but part of this had accumulated during the previous year. An urgent appeal was made for increased subscriptions, and the result of this appeal was that a sum of £1,083 more than that of the previous year was collected. There were 590 medical and surgical cases under treatment in the infirmary, and 574 were treated to a conclusion. Of these 490 were cured or improved, 45 dismissed, and 39 died. The average mortality was 6·7 per cent.; but if 20 cases dying within 48 hours be excluded, the mortality was only 3·4 per cent. The average residence was 39·4 days, which would be considered a long time in English hospitals. The average number resident was 63, and the greatest number in the house at any one time was 80. In the fever house 79 cases of scarlet fever were admitted. The average mortality was 4·7 per cent.· but 2 patients were moribund on admission. Of 19 cases of enteric fever 4 died, 1 being moribund on admission. Table III. shows that 163 operations were performed during the year; and this table gives ample information about the sex, numbers, whether cured or improved, and the deaths, of all patients operated on. The medical and surgical cases are given with the same completeness.

### COUNTY HOSPITAL, LINCOLN.

The total income was £5,283, and the total payments £5,052. Therefore the annual income was not only sufficient to meet the expenditure, but the committee were able to reduce the adverse balance of last year from £403 to £171. The probationers' fees amounted to £173. The daily average number resident was 95, and the number of in-patients admitted was 998. There were 83 in residence at the beginning of the year, and this gives a total of 1,081. Of these 550 were cured, 143 relieved, 49 not relieved, 186 were made out-patients, 61 died, and 88 remained in the institution. The average length of residence of each patient was 33 days, and the cost was 20s. a week. There 2,174 out-patients. The medical and surgical tables merely give the numbers suffering from the various diseases, but the table of operations shows the cause for the operation, and whether cured, relieved or died. Chloroform was administered on 366 occasions, ether on 8, and the A.C.E. mixture on 13. The governors caution the subscribers not to give recommendations to those able to pay for medical advice. It would be well for hospitals if this caution were universally given and universally acted upon.

